# Variations and Determinants of Hospital Costs for Acute Stroke in China

**DOI:** 10.1371/journal.pone.0013041

**Published:** 2010-09-28

**Authors:** Jade W. Wei, Emma L. Heeley, Stephen Jan, Yining Huang, Qifang Huang, Ji-Guang Wang, Yan Cheng, En Xu, Qidong Yang, Craig S. Anderson, for the ChinaQUEST Investigators

**Affiliations:** 1 The George Institute for Global Health, Royal Prince Alfred Hospital and University of Sydney, Sydney, Australia; 2 Department of Neurology, Peking University First Hospital, Beijing, China; 3 Centre for Epidemiological Studies and Clinical Trials, Ruijin Hospital, Shanghai Jiaotong University School of Medicine, Shanghai, China; 4 Department of Neurology, General Hospital of Tianjin Medical University, Tianjin, China; 5 Department of Neurology, The Second Affiliated Hospital of Guangzhou Medical College, Guangzhou, China; 6 Department of Neurology, Xiangya Hospital of Centre-South University, Changsha, China; Erasmus University Rotterdam, Netherlands

## Abstract

**Background:**

The burden of stroke is high and increasing in China. We modelled variations in, and predictors of, the costs of hospital care for patients with acute stroke in China.

**Methods and Findings:**

Baseline characteristics and hospital costs for 5,255 patients were collected using the prospective register-based ChinaQUEST study, conducted in 48 Level 3 and 14 Level 2 hospitals in China during 2006–2007. Ordinary least squares estimation was used to determine factors associated with hospital costs. Overall mean cost of hospitalisation was 11,216 Chinese Yuan Renminbi (CNY) (≈US$1,602) per patient, which equates to more than half the average annual wage in China. Variations in cost were largely attributable to stroke severity and length of hospital stay (LOS). Model forecasts showed that reducing LOS from the mean of 20 days for Level 3 and 18 days for Level 2 hospitals to a duration of 1 week, which is common among Western countries, afforded cost reductions of 49% and 19%, respectively. Other lesser determinants varied by hospital level: in Level 3 hospitals, health insurance and the occurrence of in-hospital complications were each associated with 10% and 18% increases in cost, respectively, whilst treatment in a teaching hospital was associated with approximately 39% decrease in cost on average. For Level 2 hospitals, stroke due to intracerebral haemorrhage was associated with a 19% greater cost than for ischaemic stroke.

**Conclusions:**

Changes to hospital policies to standardise resource use and reduce the variation in LOS could attenuate costs and improve efficiencies for acute stroke management in China. The success of these strategies will be enhanced by broader policy initiatives currently underway to reform hospital reimbursement systems.

## Introduction

In China, the world's most populous country, stroke is an enormous health care burden, representing the second leading cause of death and occurring in approximately 2 million people each year [1]. In light of demographic shifts towards an older and more urbanised population, and the limited available treatment and prevention strategies, the high costs of stroke care are projected to rise in China.

Two key dimensions to the economic burden of major chronic diseases such as stroke, in many developing countries such as China are: the lack of universal health insurance coverage so that significant cost impacts are borne directly by patients and their families [2]; and increasing health care costs associated with investigations, medications and treatment. These factors, compounded by uncertainties over the overall costs of treatment at the onset of illness, are likely to impair access to timely care. Recent efforts to expand the coverage of social health insurance to urban and rural regions in China, has great potential to encourage access to care and reduce the economic impact of illness and, despite some early mixed results [3]–[6], appears to be producing positive outcomes [7]. Another initiative, case-based hospital funding that was introduced in 2007, is expected to promote uniformity in the costs of hospital care, with 22% of Chinese hospitals already adopting this method of payment [8].

A clear understanding of the drivers of variations in health care costs is instrumental to developing and implementing policies aimed at improving the efficiency of public expenditure. At present, inflationary pressures in the Chinese health system have been exacerbated by a reimbursement system that is essentially fee-for-service and linked to provider remuneration which in turn can create incentives for unnecessary care [9]–[11]. However, proposed pilot studies of innovative provider payment systems offer promising mechanisms to address this problem [10].

Although data on the economic aspects of stroke using a mix of prevalence and incidence based approaches are widely available [12]–[17], the few studies undertaken in China [18]–[21] have been restricted in location and the assessment of costs, thereby limiting their ability to capture the full range of variation and key determinants of costs. We aimed to examine factors associated with variation in the hospital costs of stroke by including data from hospitals in varied geographical and socioeconomic regions of China.

## Methods

### Ethics statement

The study was approved by the ethics committees of Peking University First Hospital (Beijing), Ruijin Hospital (Shanghai), Prince of Wales Hospital (Hong Kong), and The University of Sydney. Good Clinical Practice guidelines in accordance with the Declaration of Helsinki were used, and the privacy of patients was strictly protected.

### Study design

The ChinaQUEST (QUality Evaluation of Stroke Care and Treatment) study used a prospective, national, multi-centre, hospital-based register to collect data on patients admitted to hospital with acute stroke across mainland China over a 5-month period in 2006. Chinese hospitals are classified as Level 1 (community hospitals with only the most basic facilities and very limited inpatient capacity), Level 2 (hospitals with at least 100 inpatient beds providing acute medical care and preventative care services to populations of at least 100,000), and Level 3 (major tertiary referral centres in provincial capitals and major cities). Of the 70 Level 2 and 3 hospitals that were invited to participate, 62 hospitals in 37 cities agreed and completed the study requirements, as described in detail elsewhere [2]. Briefly, the study included consecutive patients (age ≥15 years) with stroke due to cerebral infarction (‘ischaemic stroke’) or intracerebral haemorrhage (ICH) who agreed to 4 interview-based assessments at: baseline (after admission), hospital discharge, and 3 and 12 months follow-up. Written informed consent was obtained from all patients, or an appropriate family member (in situations where the patient was disabled) to participate.

Information on baseline sociodemographic situation was obtained predominantly by face-to-face interviews, in-hospital details were obtained through medical records and interviews with patients or proxies, and follow-up details including health expenditure were obtained primarily through telephone interviews. Baseline information collected included patient socio-demographic characteristics, clinical features, and history of co-morbid cardiovascular (CV) risk factors. Pathological stroke type, diagnostic and management options employed in-hospital, and various outcome measures of patients on discharge, were also recorded. Data were transferred to electronic Case Record Forms on a secure website connected to a central, password-protected, database located at The George Institute for Global Health in Sydney, Australia. Lead investigators at participating hospitals were also asked to complete a site questionnaire detailing specific hospital information and unit costs of medications, investigations, procedures, and ward costs, at their hospital to ascertain a list of itemised cost for their facility.

### Explanatory and outcome variables

All the costs of in-hospital care for stroke (N = 5,255) were obtained from patients (or proxies) who were asked to estimate their overall expenditure for hospital treatment at the time of the 3 month follow-up assessment. As patients are generally given an invoice of itemised hospital costs at the time of hospital discharge, these data were expected to be well recorded and to provide a reliable outcome measure. The following covariates were included *a priori* in multivariate models based on evidence of their associations with hospital utilisation: age [22], gender [23]–[24], marital status [25], living alone [26], owns health insurance [19], CV risk [19], [22], [25], pathological stroke type [24], [27], severe Glasgow Coma Scale (GCS) score on admission (defined as 3–8 of a top score of 15) [22], [28]–[29], received assisted feeding in-hospital [30], experienced any in-hospital complication [19], [24], length of hospital stay (LOS) [19], [22], and hospital characteristics [23] including size, teaching status and location. Annual household income was also included as a covariate, given the likely association with health insurance. In addition, disability/dependency on discharge (defined by a modified Rankin Scale [mRS] score of 3–5) was also included to account for stroke severity on recovery.

CV risk was modelled as a continuous measure, graded from 0 to 10 according to the number of CV co-morbid risk factors present, based either on: (i) responses to questions about prior history “Before this stroke, was the patient ever told they have any of the following: high blood pressure, a previous stroke, diabetes mellitus, high cholesterol/lipids, atrial fibrillation (AF), previous TIA (transient ischaemic attack), heart attack/myocardial infarction, angina/coronary heart disease”, or: (ii) new diagnoses of AF, coronary heart disease, hypertension, diabetes mellitus, and hyperlipidaemia/elevated cholesterol (including patients with total cholesterol level ≥5.20 mmol/L on discharge [31]) reported post-stroke in-hospital. Cigarette smoking, regular alcohol consumption within the 3 months before the stroke, and being overweight (based on a body mass index ≥24 kg/m^2^ [31]) were also included in the CV risk assessment. In-hospital complication was defined as pneumonia, deep venous thrombosis, recurrent stroke, urinary tract infection, other sepsis, pulmonary embolus, coronary event, seizure, fall with injury, or any other clinically significant event that prolonged hospital stay. Location of the hospital was considered according to: (i) economic factors, with the 2006 per capita Gross Regional Product (GRP) for the province in which the hospital is located and; (ii) geography, with hospitals segregated into ‘northern’ (includes the areas of Beijing, Hebei, Heilongjiang, Henan, Inner Mongolia, Jilin, Liaoning, Qinghai, Shaanxi, Shandong, Tianjing, and Xinjiang) and ‘southern’ (includes the areas of Anhui, Chongqing, Fujian, Guangdong, Guangxi, Hubei, Hunan, Jiangsu, Jiangxi, Shanghai, Sichuan and Yunnan province).

### Statistical analyses

Data excluded from these analyses were those from patients (n = 50) registered in Hong Kong due to the city having different economic and healthcare patterns to mainland China. Significance of baseline differences between included and excluded patients, were assessed using χ^2^ test for categorical variables, *t* test for continuous normally distributed variables, and Mann Whitney *U* test for continuous skewed variables. To account for non-negativity and positively skewed empirical distribution of costing data, several statistical models have been evaluated including ordinary least squares (OLS) and generalised linear models (GLM) with log links: GLM models generally produce more consistent models albeit with imprecise estimates if the log-scale error is heavy-tailed; whilst OLS models can be biased and inefficient when heteroskedasticity exists [32]–[33]. In these analyses, the log-scale residuals from the GLM models were heavily-tailed (coefficient of kurtosis >20 regardless of the specific GLM model used, γ Gaussian or Poisson) and there was no evidence of heteroskedasticity in the OLS model whether assessed visually or by using Koenker's N*R2 version of the score test (which does not assume normally distributed errors). Thus, we initially selected the OLS model based on the Manning and Mullahy checklist [32]. In addition, although a random effects model estimated using feasible generalised least squares may have been more efficient, the Hausman specification test was violated, so a random effects model would have produced biased estimators [34]. Therefore, for this study, OLS regression analysis was conducted to determine factors associated with in-hospital cost (converted to US$100 to facilitate interpretation of coefficients) with prior log transformation of model outcome due to skewed raw data. Furthermore, to account for clustering of patients at the hospital level, clustered standard errors were estimated [33]. Age, number of CV risk factors, GRP and log transformed LOS were included as continuous variables. Checks were made of all plausible interactions and collinearity between variables. Collinearity was assessed by examination of bivariate scatterplots, correlation matrix, mean and individual variance inflation factors, and conditional numbers. As collinearity was detected between the level and size of hospitals, models were developed separately for Level 2 and 3 hospitals. All significant interactions were initially identified. Backward stepwise approach was then used to successively remove non-significant interactions in the full models.

Sensitivity analyses were conducted to examine the effect of: (i) excluding patients with any re-admission after the initial hospital stay; and (ii) imputing by chained equations [35] missing values of cost, LOS, and GCS and mRS scores using model covariates as well as the following auxiliary variables: education level; employment status; occupation type; prior dependency; Oxfordshire Community Stroke Project classification of stroke; death by 3 months post-stroke; time from symptom onset to hospital presentation (log transformed); use of the following intravenous therapies in hospital: thrombolysis, corticosteroid, haemodiluting agents (such as mannitol), neuroprotectants (including edaravone, ganglioside GM1, cattle encephalon glycoside and ignotin, cinepazide, citicholine), traditional Chinese medicine (TCM, alternative/herbal medicine); and use of antihypertensive, antiplatelet and lipid lowering therapies in-hospital and during the 3 months post-stroke.

In addition, to determine the full extent of the effect of modifiable variables on cost, their marginal effects by hospital level and stroke type (i.e. holding all other variables constant at their means) were calculated from coefficients derived from the model. As residual variance was not normally distributed but homoskedastic, Duan's non-parametric retransformation [36] factor (1.24 for Level 3 hospitals, 1.25 for Level 2 hospitals) was used in the evaluation of the marginal effects. Moreover, one-way analysis of variance (ANOVA) was performed to determine the proportion of variance associated with differences in cost amongst hospitals by hospital level. Itemised costs were based on investigator provided hospital data. Statistical significance was considered at p<0.05. All analyses were conducted using STATA 10.1 (StataCorp LP, College Station, TX).

## Results

**Figure 1:**
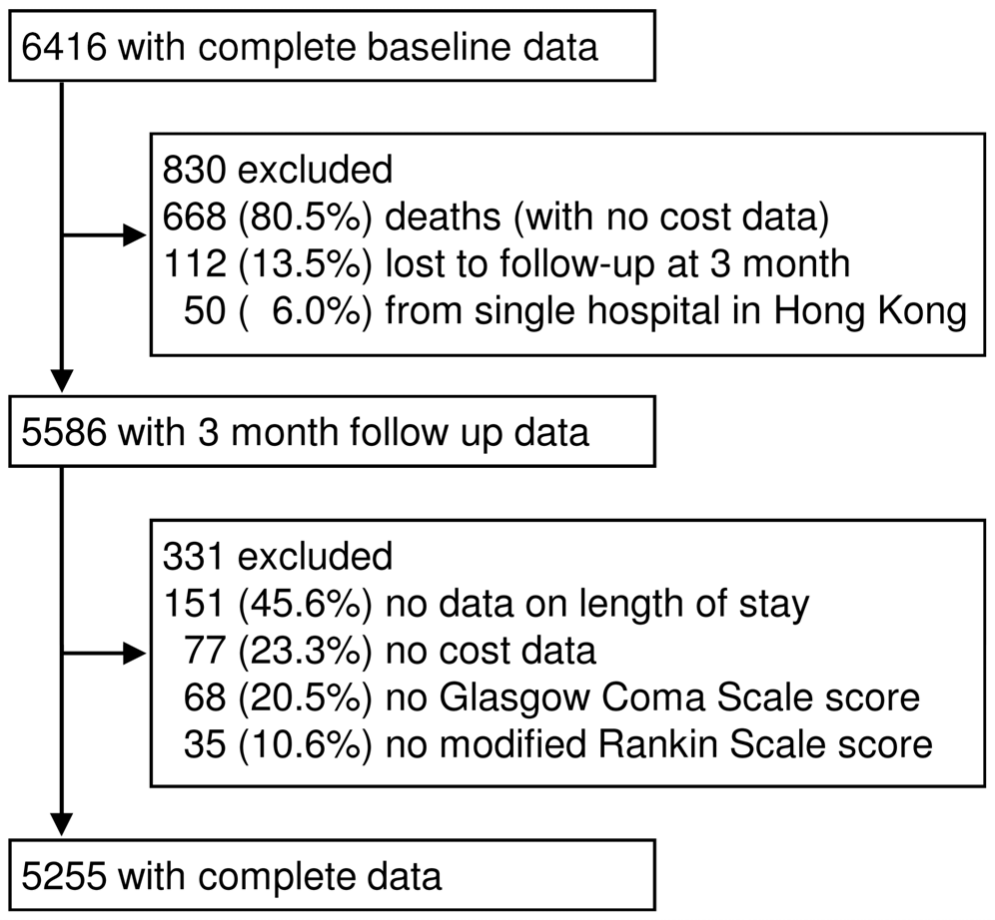
Patient flow.

Figure 1 shows the flow of patients. Of 6,416 patients with baseline data, 1,161 were excluded due to death and no cost data (n = 668), or missing data and *a priori* exclusion criteria (n = 493). Thus, there were 5,255 patients with complete data for analyses. Compared with patients excluded due to death and no cost data, those included in analyses were more likely to be younger, married, have health insurance, higher incomes, stroke due to lacunar infarction, longer LOS, and to be managed in large Level 3 hospitals. In addition, fewer included patients had ICH, large artery cerebral infarcts, severe GCS score on admission, in-hospital assisted feeding and complications, and death/disability at discharge (Table S1). Separate comparison of the included patients with those excluded for reasons other than death (n = 493) showed similar distributions of age, gender, CV risk profile, initial severity, in-hospital assisted feeding/complications, LOS, disability on discharge, and geographical location of admitting hospital. However, included patients were more likely to be married, not living alone, not insured for health, a low income earner, an ICH patient, surviving after hospital discharge, to have lower CV risk, and to have been managed in a Level 2 non-teaching hospital in a low GRP area (data available on request).

Table 1 shows the costs of selected common investigations, treatments and wards for patients with stroke, based on hospital supplied itemised costs. The highest reported costs were for magnetic resonance imaging (MRI) with contrast media, intravenous tissue plasminogen activator (tPA), intravenous neuroprotectant ganglioside GM1, and care in an intensive care unit (ICU). As shown by the high coefficients of variation, the greatest variations in cost between hospitals were for the use of calcium channel blockers and ICU stay in Level 3 hospitals, and defibrase and TCM in Level 2 hospitals.

**Table 1:** Average cost of selected investigations, treatments, and wards, by hospital level in China.

			Level 3 hospital (N = 45)		Level 2 hospital (N = 14)	
			Mean (Median) CNY	C_v_ *	Mean (Median) CNY	C_v_ *
*Investigations (by single test)*						
	Computerised tomography, brain (no contrast)		262 (250)	0.41	195 (180)	0.29
	Magnetic resonance imaging, brain (+ contrast)		1053 (1035)	0.29	750 (730)	0.54
	Electrocardiogram		23 (20)	0.43	19 (20)	0.34
	Doppler ultrasound of carotids		172 (170)	0.32	158 (140)	0.50
	Transthoracic echocardiogram		191 (200)	0.45	174 (200)	0.24
	Transcranial doppler ultrasound		132 (120)	0.41	93 (94)	0.43
*Treatments – intravenous (by ampoule)*						
	tPA (tissue plasminogen activator)		5226 (6283)	0.53	7843 (7843)	0.21
	Urokinase		248 (230)	0.80	279 (220)	0.78
	Defibrase		315 (314)	0.61	273 (80)	1.84
	Mannitol		11 (6)	1.66	5 (4)	0.36
	Neuroprotectants					
		Edaravone	211 (210)	0.46	289 (237)	0.34
		Ganglioside GM1	465 (340)	0.78	373 (160)	0.99
		Cattle encephalon glycoside & ignotin	186 (200)	0.59	262 (99)	1.38
		Cinepazide maleate	129 (116)	0.48	74 (67)	0.38
		Citicholine	8 (4)	1.30	5 (4)	0.50
	Traditional Chinese medicine		66 (59)	0.66	52 (46)	0.75
*Treatments – oral (by tablet)*						
	Beta blocker		2 (1)	2.27	1 (1)	0.69
	Calcium channel blocker		7 (3)	3.89	2 (1)	1.05
	Angiotensin-converting enzyme inhibitor		3 (2)	1.50	1 (1)	1.24
	Clopidogrel		22 (21)	0.48	19 (22)	0.25
	Angiotensin II receptor blocker		6 (6)	0.48	5 (6)	0.50
	Traditional Chinese medicine		7 (3)	1.25	11 (8)	1.42
*Wards (per day)*						
	General medical ward		44 (40)	0.55	35 (30)	0.60
	Neurology ward		49 (38)	0.67	40 (32)	0.68
	Intensive care unit		375 (200)	1.66	179 (140)	0.72

*Coefficient of variation, equal to the standard deviation divided by the mean; to convert CNY to US dollars, divide by 7.

**Figure 2:**
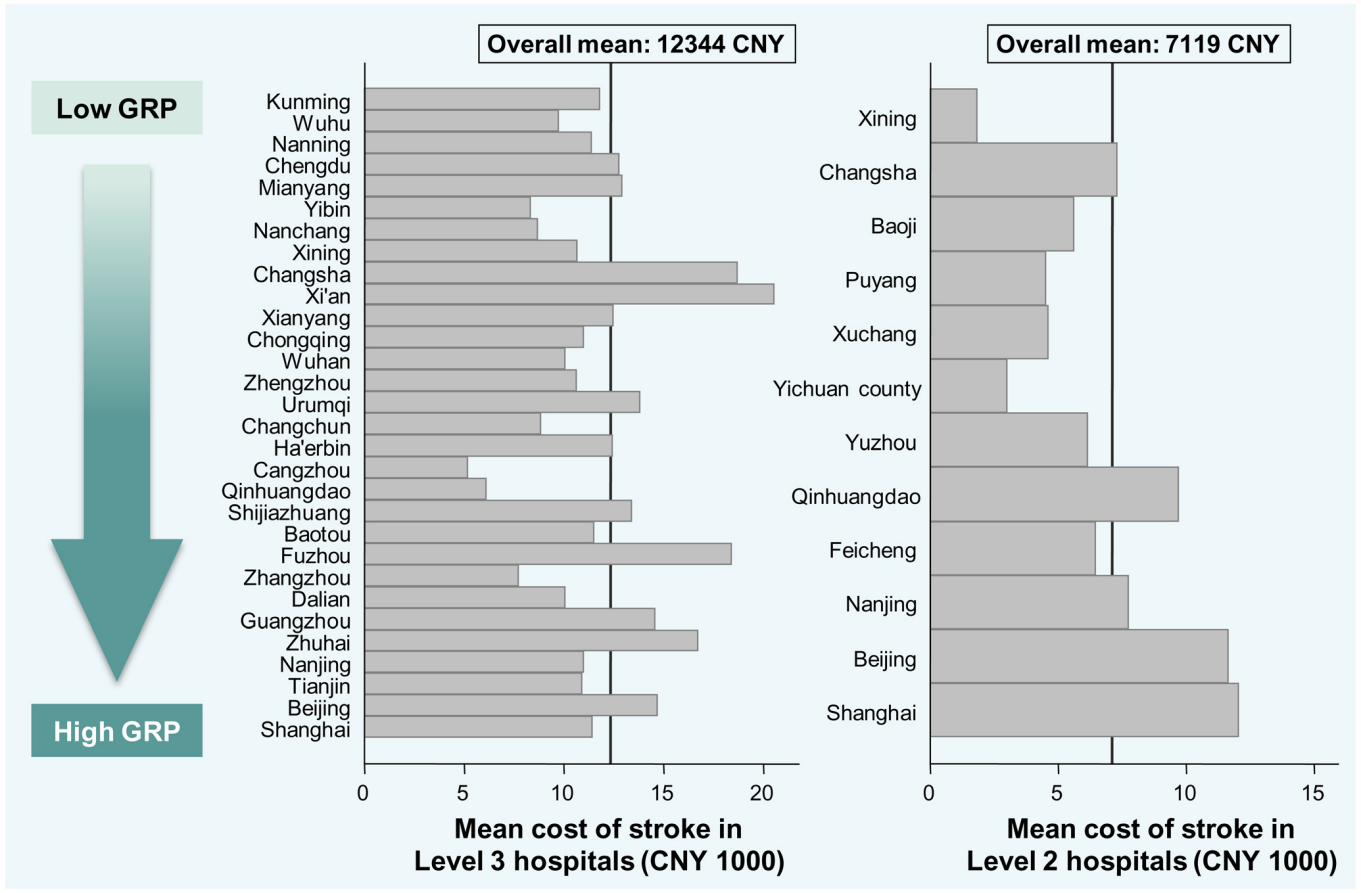
Mean total cost of stroke, by hospital type, location and 2006 per capita GRP. GRP, Gross Regional Product for the province.

**Figure 3:**
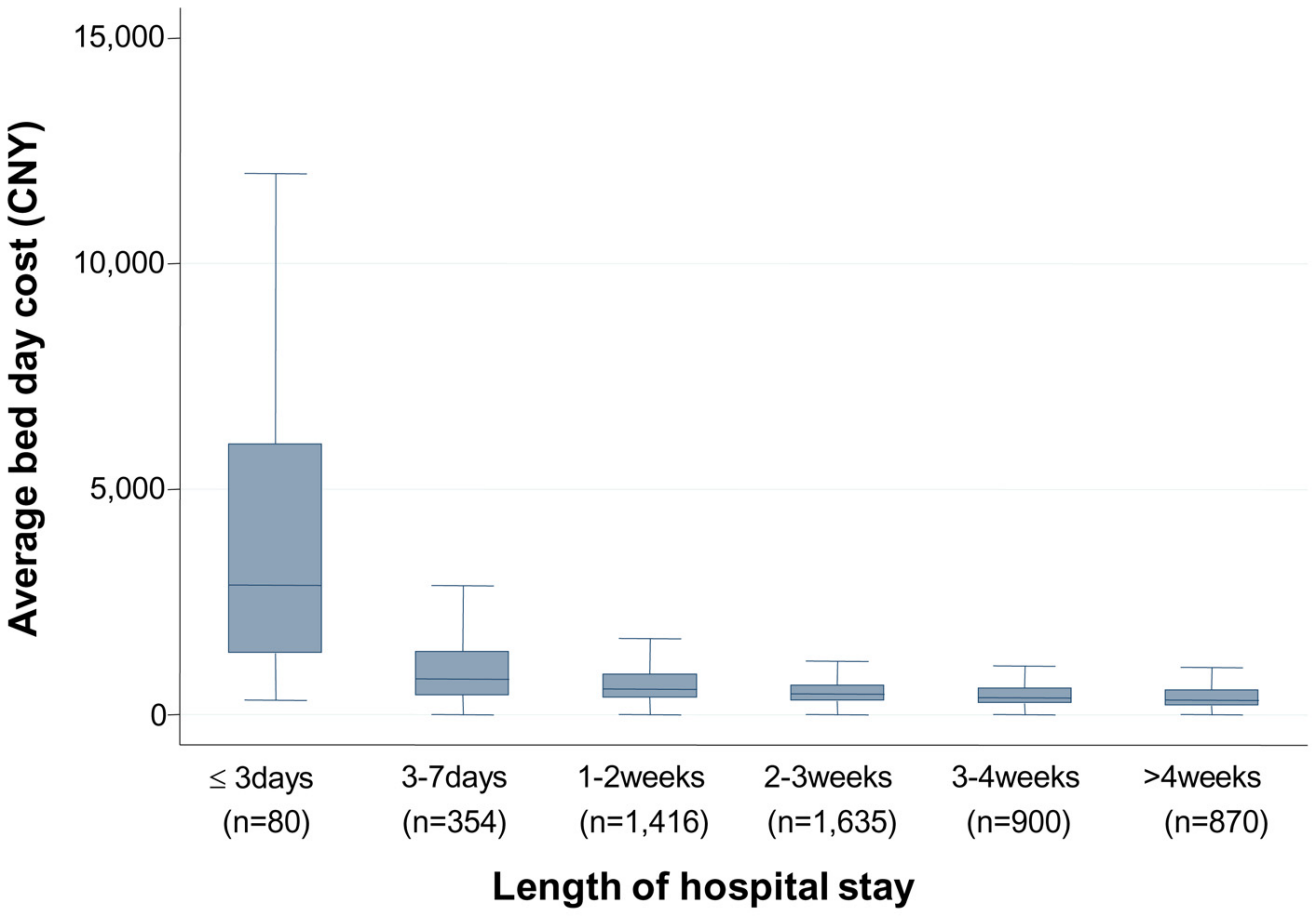
Box plots of average bed day cost by length of stay. CNY denotes Chinese Yuan Renminbi, with 7 CNY equating to one United States dollar; the central line denotes the median, the box encompasses the 25^th^ and 75^th^ percentiles, and the whiskers denote the lower and upper adjacent values as defined by Tukey [47].

Overall average LOS was 20 days (median 17, interquartile range 12–25), but varied by hospital level (Level 3 20 days, Level 2 18 days) and stroke type (ischaemic stroke 19 days, ICH 23 days). The mean cost of acute stroke care was 11,216 CNY (≈US$1,602) per patient, but this figure differed widely by the location and level of hospital (Figure 2), and was lower for ischaemic stroke (10,689 CNY; ≈US$1,527) than ICH (13,089 CNY; ≈US$1,870). Mean daily cost of stroke was 565 CNY (≈US$81) per person overall (based on mean LOS), and was 565 CNY (≈US$81) and 564 CNY (≈US$81) per person for ischaemic stroke and ICH, respectively. In addition, cost varied by LOS: median average bed day cost was CNY 2,883 (≈US$412) for patients who stayed ≤3 days in hospital and CNY 333 (≈US$48) for patients with LOS of >4 weeks (Figure 3).

Multivariate regression analysis showed that factors significantly associated with increased cost of acute stroke care for Level 3 hospitals included: younger age, being married, having health insurance, higher household income, stroke due to large artery cerebral infarction, greater initial stroke severity, need for assisted feeding, presence of in-hospital complications, long LOS, being disabled/dependent on discharge, and being treated in a large hospital in a rich province (Table 2). Notably, having health insurance, high annual household income (≥20,000 CNY [≈US$2,857]), a large artery cerebral infarction, and in-hospital complication(s) were each associated with 10%, 30%, 11%, and 18% increases in cost, respectively. Similarly, for Level 2 hospitals, marital status, assisted feeding, LOS, and disability on discharge, were associated with increased costs. In addition, ICH was associated with a 19% increase in cost. Model forecasts further illustrate the impact of these variables on cost, in particular for LOS, where reduction to a fixed LOS of 1 week, that is typical for stroke in hospitals of Western countries, affords approximately 49% and 19% reductions in costs for Level 3 and 2 hospitals, respectively (Table 3). Finally, 8% and 19% of the variation in cost in Level 3 and Level 2 hospitals, respectively, were attributable to inter-hospital differences.

**Table 2:** Factors associated with total cost of stroke in hospital in China*.

Predictor			Level 3 hospital (N = 4,120)			Level 2 hospital (N = 1,135)		
			Regression coefficient	Standard error	P-value	Regression coefficient	Standard Error	P-value
*Sociodemographic*								
	Age: per year		−0.003	0.001	0.02	0.002	0.002	0.33
	Female		0.019	0.021	0.37	−0.030	0.048	0.54
	Married		0.499	0.165	0.004	0.128	0.059	0.05
	Living alone		−0.032	0.065	0.63	−0.037	0.117	0.75
	Owns health insurance		0.106	0.048	0.03	−0.082	0.076	0.30
	Annual household income†							
		10,000 – 19,999 CNY (≈ US$1,429–2857)	0.322	0.059	<0.001	−0.145	0.126	0.27
		≥20,000 CNY (≈ US$2,857)	0.362	0.084	<0.001	0.249	0.134	0.09
		Declined to respond/unknown	0.022	0.074	0.76	−0.058	0.071	0.43
*Medical/clinical features*								
	Cardiovascular risk factors: per risk factor		0.007	0.008	0.37	0.025	0.021	0.26
	Stroke type‡							
		Large artery cerebral infarct	0.113	0.053	0.04	−0.065	0.068	0.36
		Intracerebral haemorrhage	0.035	0.036	0.33	0.208	0.073	0.01
		Other/uncertain	0.098	0.056	0.09	−0.092	0.168	0.59
	Severe GCS score on admission		0.158	0.064	0.02	0.198	0.097	0.06
	Assisted feeding in-hospital		0.352	0.081	<0.001	0.269	0.122	0.05
*Outcome at discharge*								
	Experienced ≥1 in-hospital complication		0.198	0.049	<0.001	−0.0300	0.073	0.69
	Length of hospital stay§		0.636	0.068	<0.001	0.227	0.050	0.001
	Disability/dependency		0.252	0.036	<0.001	0.242	0.067	0.003
*Hospital characteristics*								
	Large size (>500 beds)		0.610	0.191	0.002	−0.137	0.232	0.56
	Teaching		−0.502	0.09	<0.001	0.117	0.324	0.72
	Situated in northern China		−0.008	0.085	0.93	−0.282	0.262	0.30
	GRP∥: per US$100		0.009	0.003	0.001	0.013	0.007	0.10
*First order interactions*								
	Severe GCS score on admission x assisted feeding		0.269	0.106	0.01	-	-	-
	In-hospital complication x disability/dependency		−0.121	0.052	0.02	-	-	-
	Length of stay x married		−0.137	0.059	0.03	-	-	-
	Length of stay x large hospital size		−0.216	0.065	0.002	-	-	-
	Large hospital size x teaching hospital		0.459	0.109	<0.001	-	-	-
	Health insurance x GRP∥		−0.003	0.001	0.03	-	-	-
	Health insurance x income 10,000 – 19,999 CNY		-	-	-	0.264	0.116	0.04
	GRP∥ x income 10,000 – 19,999 CNY		−0.008	0.002	<0.001	-	-	-
	GRP∥ x income ≥20,000 CNY		−0.006	0.002	0.005	-	-	-

*Model outcome is log transformed, unit is US$100.

† Reference group: annual household income ≤ 9,999 CNY (≈ US$1,428).

‡ Reference group: small artery lacunar infarct; Other/uncertain category includes: cardioembolic, retinal, venous and other defined infarcts, infarcts of unknown cause and stroke of uncertain pathological type.

§ Variable were further log transformed before regression.

∥ 2006 per capita gross regional product for province in which hospital is located.

**Table 3:** Predicted cost of stroke by stroke type and modifiable variables for Level 2 and 3 hospitals*.

Variable		Small artery lacunar infarct		Large artery cerebral infarct		Intracerebral haemorrhage	
		Predicted cost (CNY)	Potential saving† (CNY)	Predicted cost (CNY)	Potential saving† (CNY)	Predicted cost (CNY)	Potential saving† (CNY)
*Level 3 hospital*							
	Experienced ≥1 in-hospital complication	12,005	2,156	16,227	2,913	14,721	2,643
	Disability/dependency at discharge	12,189	2,715	15,463	3,444	14,226	3,169
	Length of stay: mean‡ vs. 7 days	10,958	5,069	15,325	7,942	14,061	7,606
	Non-teaching hospital	15,667	6,179	22,338	8,810	19,819	7,817
*Level 2 hospital*							
	Disability/dependency at discharge	6,931	1,489	7,154	1,537	8,299	1,783
	Length of stay : mean vs. 7 days	6,076	1,042	6,799	1,283	7,956	1,779

*To convert CNY to US dollars, divide by 7.

† For in-hospital complication, disability/dependency and non-teaching hospital: potential saving = predicted cost for average person with variable – predicted cost for average person if variable was removed, holding all variables constant at their means; for length of stay: potential saving  =  predicted cost for average person with mean length of stay – predicted cost for average person if length of stay was reduced to 7 days, holding all variables constant at their means.

‡ 19 days, 22 days, and 24 days for small artery lacunar infarct, large artery cerebral infarct, and intracerebral haemorrhage patients, respectively in Level 3 hospital.

§ 16 days, 18 days, and 21 days for small artery lacunar infarct, large artery cerebral infarct, and intracerebral haemorrhage patients, respectively in Level 2 hospital.

Sensitivity analyses excluding those with hospital re-admission(s) post-discharge (n = 526) showed that health insurance, income, LOS and hospital characteristics remained unchanged as the key drivers of cost. For Level 2 hospitals, the main effects remained essentially unchanged following exclusion of those patients with hospital re-admissions; however the interaction between health insurance and income became non-significant. Sensitivity analyses using multiple imputations to account for biases that may have resulted from missing values of cost, GCS, mRS and LOS showed that for Level 3 hospitals, results were unchanged apart from a few interactions that became no longer significant. In analyses of imputed data for Level 2 hospitals, marital status and the interaction between insurance and income became non-significant, while patients with severe GCS score on admission were found to have increased costs.

## Discussion

This large national health outcome study has documented the various costs of hospital care that may be associated with an episode of acute stroke in China. We estimated that, on average, the overall hospital cost for stroke is 11,216 CNY (≈US$1,602), which equates to 565 CNY (≈US$81) per day according to an average LOS of nearly 3 weeks (20 days). Given a national average annual wage of 21,001 CNY (≈US$3,000) in 2006 [37], this health care cost translates into more than half a year's wage for many people in China. These estimates are higher than the official (mean hospital cost) figures from the Chinese Ministry of Health of 6,325 CNY for ischaemic stroke and 7,732 CNY for ICH [38]. This may have been due to many of the patients in our study having long LOS, being included mainly from urban locations, and for any hospital re-admission costs within the 3 month follow-up period to have been included in the estimates. Even so, our cost estimates are comparable with those reported in a 2 centre, single city, study by Gao *et al* [21] in China, and in the findings of lower costs for ischaemic stroke than ICH as has been shown in studies from Greece [27], United States [39], Australia [40], and Japan [41].

Variations in the cost of acute stroke tend to be driven by stroke severity and hospital LOS, with patients with more severe neurological impairment (lower GCS scores) on admission, requiring assisted feeding, being more disabled (mRS) at discharge, and having longer LOS, being more likely to incur higher costs of care regardless of the level of hospital. In Level 3 hospitals, though, amount of household income and possession of health insurance were also positive drivers of cost, suggesting the potential for discriminatory pricing by socioeconomic status. Another discrepancy noted between the varying hospital levels was that in-hospital complications were predictive of cost variation in Level 3 but not Level 2 hospitals, raising questions about the potential higher risks of infectious and other complications in larger hospitals that could impact on LOS, outcomes and costs. Indeed, we found that Level 3 hospitals had a higher frequency of infectious complications (including pneumonia, urinary tract infection, sepsis) than Level 2 hospitals (13% vs. 6%). These findings are analogous to previous studies in China which have also reported LOS, health insurance status, infection during hospital stay, and stroke severity, all being strongly associated with increased total inpatient cost [18]–[21]. Conversely, a clear explanation for why age was associated with reduced costs in Level 3 hospitals but not Level 2 hospitals, is not readily forthcoming. However, there may be differences in the type and level of intervention (and care) being assigned to those patients with a poor prognosis between these types of hospitals. Interestingly, we did not find gender to be associated with variation in costs at either hospital level, which is consistent with a recent study in Greece [27], but differs from Evers *et al* [24] who found that females were more likely to experience higher costs than males. Such comparisons between studies, however, are likely confounded by differences in case-mix and patterns of care. Moreover, ICH was not a predictor of cost variation in Level 3 hospitals, a finding which may be due to shorter LOS (and/or more conservative management) for a condition which tends to have a worse prognosis than ischaemic stroke. In addition, patients with multiple CV co-morbidities/risk factors were not shown to be subjected to higher cost, irrespective of hospital level, however, this is consistent with the current literature where both significant and null results have been reported for associations between CV co-morbidities and hospital utilisation [22].

Our study also found that costs tended to be higher in large Level 3 hospitals located in provinces with a high GRP. This may be indicative of increased likelihood of severe and complex patients being managed in larger tertiary referral hospitals, and higher staff and infrastructure costs for hospitals in more affluent provinces. Among the Level 3 hospitals, though, those with teaching hospital status had markedly lower costs, which may reflect educational and awareness activities to promote best practice, evidence based guidelines and clinical care pathways. In addition, reduced variation in cost between Level 3 hospitals and Level 2 hospitals may reflect a higher proportion of teaching hospitals in the former focussed on evidence based practice and with less potential for discriminatory pricing.

Given that LOS is a main determinant of hospital costs, the adoption of case based funding through the use of Diagnosis Related Group (DRG) Prospective Payment Systems, as has been implemented in the United States [42], Australia and other countries, offers promise in reducing health care costs in China. For example, an Australian study has shown that the direct and indirect costs incurred during the first year after stroke (excluding inpatient costs for the primary event and average LOS in hospital, and where average LOS was about 1 week) were: on average, Australian [AU] $19 per person per day for ischaemic stroke and AU $28 for ICH; whereas, average daily hospital costs (calculated from average LOS) were AU $490 and $702 for ischaemic stroke and ICH, respectively [40]. In-hospital medication use, notably the high use of non-evidence based therapies such as neuroprotectants (e.g. edaravone, ganglioside, etc) and TCM, may also contribute to the burden of health costs in China, with previous studies and national estimates revealing that more than half the average cost of hospitalisation is composed of medication costs for both ischaemic stroke and ICH [20]–[21], [38]. The implementation of clinical care pathways may assist in standardising care, thereby reducing the use of non-evidence based therapy and minimising variation in LOS in acute stroke [43].

We recognise our study has limitations that deserve comment. Firstly, we were unable to examine the effect of other factors which may have influenced hospital costs, including physician characteristics and staff capacity. Secondly, our study was predisposed to survivor bias, as cost estimates could only be obtained for a small proportion of patients who had died. However, patients experiencing early death after stroke are likely to have lower costs due to greater severity and/or co-morbidities, and shorter hospital LOS. In addition, the characteristics of such patients were likely captured to some extent amongst the cost driver (predictor) variables included in the models. There is also the potential for differential bias, as we only had costing data available for a small number of patients who had died. However, sensitivity analyses using multiple imputations showed minimal changes to our estimates, so the effect of this bias is likely to be negligible. Thirdly, we may have over-estimated the short-term cost savings that could be derived from reducing average LOS in China, as most of the costs of care are incurred within the first few days of admission to hospital, as shown in Figure 3. Nonetheless, as stroke is a severe disease and patients are often transferred from smaller to larger hospitals for further assessment and management, it seems reasonable to assume that our cost estimates are widely applicable to patients with stroke in hospitals located in inner and peripheral, urban areas. In addition, since patients with mild-to-moderate levels of residual disability are likely to be discharged home after having most investigations and acute treatments completed within the first week of admission to hospital, the ongoing costs of hospital care are likely to be driven principally by those associated with a bed day stay. Nevertheless, efforts to reduce LOS, through for example early supported discharge, could provide the opportunity for greater service options for patients (and providers) and the potential for improved health outcomes [44]. Finally, our estimates of cost may have been subject to recall and interview bias, but given the standardised approach to data collection, we do not anticipate this to have been a major issue. Indeed, non-response to the cost item was only 1% among survivors in the study. In addition, the effect of recall or ‘social desirability’ bias is also expected to be minimal given the fee-for-service nature of the health care system in China, such that all costs are invoiced to patient or nominated family member. Although different methods of interview administration (face-to-face vs. telephone) were employed for our participant surveys, previous research has indicated that while each approach may engender some degree of social acceptability bias, the difference between modes is likely to be small [45] and the reliability of reported health behaviours high regardless of interview mode [46]. Finally, although we may have included the costs associated with early re-admission to hospital in our estimates, sensitivity analyses revealed minimal effect of excluding such patients, and the consistency of our findings with national estimates and other studies in China, provides reassurance to the validity of these data.

In summary, this study provides estimates of the current hospital costs of acute stroke care in China, and of the factors that determine variation in these costs. The LOS in hospital and background economic situation of the patient, defined either by the possession of health insurance or amount of household income, are key drivers of these health care costs, as shown in other countries with fee-for-service funding schemes. Reductions in hospital costs could result from new operational policies that include clinical pathways and standardised use of resources, coupled with improved physician training and the introduction of new models of care to reduce LOS, but this will need confirmation in future research. Our study suggests that there is scope to improve efficiencies in the management of patients with stroke through reducing LOS in some hospitals. Nevertheless, any such changes can only be sustained through broader policy development, such as those being tested in recent initiatives to introduce case-based funding and in pilot studies to reform payment systems to address misalignment of incentives and encourage appropriate treatment. Given the projected increase in the number of patients with stroke associated with the rapid aging of the population, efforts to improve the efficiency of hospitals and community services are needed at the individual hospital level but also, and arguably more importantly, through effective policies that enable reform across the entire health system.

## Supporting Information

Table S1 Baseline characteristics of patients*. *Values are reported as mean±SD, median (IQR), or number (percentage) of subjects; percentages are based on non-missing values. †Defined as ≥2 of the following: history of hypertension, diabetes, hyperlipidaemia, atrial fibrillation; prior stroke, prior transient ischaemic attack, prior coronary artery disease, cigarette smoking, regular alcohol consumption, and being overweight (body mass index ≥24 kg/m2 [31]). ‡Other/Uncertain category includes: cardioembolic, retinal, venous and other defined infarcts, infarcts of unknown cause and stroke of uncertain pathological type. ∥GCS, Glasgow Coma Scale, severe score ≤8 in range 3 (low) to 15 (high, normal). #Disability/dependence defined as modified Rankin Scale (mRS) score between 3–5. **2006 per capita gross regional product for province in which hospital is located; to convert to US$, divide by 7.
(0.05 MB DOC)
