# Supplementary material for: Variations and Determinants of Hospital Costs for Acute Stroke in China
Source: PLoS One. 2010 Sep 28;5(9):e13041. doi: 10.1371/journal.pone.0013041 (PMC2946911; doi:10.1371/journal.pone.0013041)
Supplement: Table S1 — Baseline characteristics of patients*. *Values are reported as mean±SD, median (IQR), or number (percentage) of subjects; percentages are based on non-missing values. †Defined as ≥2 of the following: history of hypertension, diabetes, hyperlipidaemia, atrial fibrillation; prior stroke, prior transient ischaemic attack, prior coronary artery disease, cigarette smoking, regular alcohol consumption, and being overweight (body mass index ≥24 kg/m2 [31]). ‡Other/Uncertain category includes: cardioembolic, retinal, venous and other defined infarcts, infarcts of unknown cause and stroke of uncertain pathological type. ∥GCS, Glasgow Coma Scale, severe score ≤8 in range 3 (low) to 15 (high, normal). #Disability/dependence defined as modified Rankin Scale (mRS) score between 3–5. **2006 per capita gross regional product for province in which hospital is located; to convert to US$, divide by 7. (0.05 MB DOC) [file pone.0013041.s001.doc]

|  | | | | | Included in the analyses (N = 5,255) | Excluded due to death & no data (N = 668) | P value |
| --- | --- | --- | --- | --- | --- | --- | --- |
| *Sociodemographic* | | | | |  |  |  |
|  | Age, mean (SD), years | | | | 63±12 | 67±13 | <0.001 |
|  | Female | | | | 2000 (38) | 264 (40) | 0.46 |
|  | Married | | | | 4437 (84) | 526 (79) | <0.001 |
|  | Living alone | | | | 235 ( 4) | 31 ( 5) | 0.84 |
|  | Owns health insurance | | | | 3198 (61) | 368 (55) | 0.004 |
|  | Annual household income | | | |  |  | <0.001 |
|  |  | | ≤ 9,999 CNY (≈ US$1,428) | | 1649 (31) | 280 (42) |  |
|  |  | | 10,000 – 19,999 CNY (≈ US$1,429 -2,857) | | 1471 (28) | 169 (25) |  |
|  |  | | ≥ 20,000 CNY (≈ US$2,857) | | 1432 (27) | 137 (21) |  |
|  |  | | Declined to respond/unknown | | 703 (13) | 82 (12) |  |
|  |  | | | |  |  |  |
| *Medical/clinical features* | | | | |  |  |  |
|  | | Cardiovascular risk factors, mean(SD)^†^ | | | 3.0±1.5 | 2.8±1.5 | <0.001 |
|  | | Stroke type | | |  |  | <0.001 |
|  | |  | | Small artery lacunar cerebral infarct | 3013 (57) | 111 (17) |  |
|  | |  | | Large artery cerebral infarct | 839 (16) | 156 (23) |  |
|  | |  | | Intracerebral haemorrhage | 1153 (22) | 345 (52) |  |
|  | |  | | Other/uncertain^‡^ | 250 ( 5) | 56 ( 8) |  |
|  | | Severe GCS score on admission^║^ | | | 262 ( 5) | 316 (48) | <0.001 |
|  | | Assisted feeding in-hospital | | | 432 ( 8) | 237 (35) | <0.001 |
|  | | | | |  |  |  |
| *Outcome at discharge* | | | | |  |  |  |
|  | Experienced ≥1 in-hospital complication | | | | 731 (14) | 284 (43) | <0.001 |
|  | Length of hospital stay (days), median (IQR) | | | | 17 (12, 25) | 6 (2, 15) | <0.001 |
|  | Disability/dependency^#^ | | | | 1979 (38) | 328 (84) | <0.001 |
|  | Death | | | | 2 (0.04) | 350 (52) | <0.001 |
|  | | | | |  |  |  |
| *Hospital characteristics* | | | | |  |  |  |
|  | Level 3 vs. Level 2 category | | | | 4120 (78) | 500 (75) | 0.04 |
|  | Large size (>500 beds) | | | | 4068 (77) | 477 (71) | 0.001 |
|  | Teaching | | | | 4462 (85) | 581 (87) | 0.16 |
|  | Situated in northern China | | | | 3044 (58) | 372 (56) | 0.27 |
|  | GRP (CNY), median (IQR)** | | | | 16962  (12457, 28814) | 16962  (12138, 28814) | 0.07 |
